# Anti‐Inflammatory Resveratrol Protects Mice From Early Mortality After Haematopoietic Stem Cell Transplantation

**DOI:** 10.1111/jcmm.70395

**Published:** 2025-02-03

**Authors:** Xiao Zhang, Wei Yu, Yimeng Sun, Xinyu Ye, Yu He, Xin Huang, Fuhao Wang, Yi Lu, Jian Zhang

**Affiliations:** ^1^ School of Medicine Southern University of Science and Technology Shenzhen Guangdong China; ^2^ Joint Laboratory of Guangdong‐Hong Kong Universities for Vascular Homeostasis and Diseases, School of Medicine Southern University of Science and Technology Shenzhen Guangdong China; ^3^ Department of Anesthesiology, the First Affiliated Hospital Jinan University Guangzhou Guangdong China; ^4^ School of Clinical Medicine Weifang Medical University Weifang Shandong China

**Keywords:** haematopoietic stem cell transplantation, inflammation, myeloid bias, resveratrol, transplant‐related mortality

## Abstract

The occurrence of inflammation subsequent to haematopoietic stem cell transplantation is associated with an elevated risk of transplant‐related mortality (TRM). However, the duration of inflammation and the potential efficacy of anti‐inflammatory agents in reducing TRM remain uncertain. We performed a comprehensive investigation to examine the post‐transplantation alterations of inflammatory mediators and to ascertain the correlation between inflammation level and TRM through the neutrophil–lymphocyte ratio, ELISAs and cytometric bead array. The findings revealed that the 30‐day interval following transplantation is characterised by the most pronounced inflammatory response in both human and murine subjects, thereby elevating the risk of TRM. The inflammation is primarily caused by myeloid bias during haematopoietic reconstitution, which is a commonly overlooked aspect in clinical transplantation, additionally, a lesser extent of irradiation‐induced injury. The administration of the anti‐inflammatory agent resveratrol has the potential to reduce systemic inflammation and TRM by suppressing the NOD‐like receptor signalling pathway and slowing down granulocyte implantation in HSCT mice. This approach did not impair the differentiation potential of haematopoietic stem cells. These findings demonstrate that the 30‐day post‐transplant period represents an opportunity to facilitate HSCT colonisation, mitigate transplant‐related adverse effects, and potentially reap the benefits of anti‐inflammatory treatments.

## 1 Introduction

Haematopoietic stem cell transplantation (HSCT) is a highly favoured treatment option for haematological disorders and malignancies as it can reinstate the haematopoietic lineage and immune system [1, 2]. Among these, haploidentical HSCT (Haplo‐HSCT) is gradually becoming the preferred method [3]. Compared to autologous HSCT and HLA‐matched sibling HSCT (MSD‐HSCT), haplo‐HSCT has a greater likelihood of causing transplant‐related mortality (TRM) [4]. TRM generally occurs within days to 2 months after clinical transplantation, and acute graft versus host disease (aGvHD) is traditionally regarded as the primary cause [5, 6]. Higher serum levels of cytokines, namely interleukin 2 (IL‐2), interleukin 17 (IL‐17), interferon‐gamma (IFN‐γ) and granulocyte‐macrophage colony‐stimulating factor (GM‐CSF), have been identified as indicators of aGvHD [7, 8, 9], indicating the potential relationship among inflammation, aGvHD and TRM. Recently, several studies have shown that various types of transplants, not only allogeneic aGvHD, can induce inflammation [10]. Alterations in cytokine levels reflect immune activation status and direct the differentiation of haematopoietic lineage cells, directly relating to the clinical outcomes.

Clinical evidences demonstrate the benefit of anti‐inflammatory immunosuppressive agents in reducing TRM, such as glucocorticoids [11, 12], lymphocyte growth inhibitors and IL‐2 receptor inhibitors [13]. However, the precise correlation between inflammation and TRM is not yet fully understood. The definite time point of inflammatory changes and whether anti‐inflammatory agents help reduce TRM, as well as the mechanisms of anti‐inflammatory agents remain uncertain. By analysing complete blood cell counts from patients undergoing allo‐HSCT, we observed a significant increase in inflammation during the initial phase after HSCT, including in patients without aGvHD. To further investigate the correlation between transplantation practice and inflammation, we established a syngeneic transplant model in mice to eliminate the effects of GvHD. We utilised cytometry by time‐of‐flight (CyTOF), RNA sequencing (RNA‐seq), and quantitative inflammation array to assess temporal alterations in systemic inflammation and haematopoietic profiles in our model. We aimed to identify the potential causes and consequences of inflammation and TRM as well as alternative therapeutic strategies.

## 2 Methods

### 2.1 Patients

Patient peripheral blood data were provided by the First Affiliated Hospital of Weifang Medical University (Weifang, Shandong, China). Inclusion criteria were as follows: diagnosis of severe aplastic anaemia (SAA) in 2010–2022 and receiving allogeneic haematopoietic stem cell transplantation (allo‐HSCT); clinical outcome was achieving complete or partial remission. Exclusion criteria were as follows: subjects with other diseases requiring radiotherapy and chemotherapy; with severe infection within the first‐month post‐transplantation; with severe liver and kidney disease. Absolute neutrophil and lymphocyte counts were collected up to 500 days after transplantation.

### 2.2 Mice

Wild‐type C57BL/6J mice (C57BL/6J^*wt*^) and transgenic C57BL/6J mice expressing enhanced green fluorescent protein (EGFP) (C57BL/6J^*egfp*^) were purchased from the Jackson Laboratory (Bar Harbour, ME, USA) were maintained in a pathogen‐free facility. All experiments were performed according to protocols approved by the Institutional Animal Care and Use Committee at the Laboratory Animal Center of Southern University of Science and Technology.

### 2.3 Total Body Irradiation‐Based and Mobilisation‐Based Transplantation

Haematopoietic stem/progenitor cells (HSPCs) were isolated from bone marrow cells using the lineage cell depletion kit (Miltenyi). The study utilised 3‐month‐old female C57BL/6J^*wt*^ mice as the recipient subjects. In the context of total body irradiation (TBI) based myeloablation transplantation (TBI‐based HSCT), the recipient mice were subjected to 10Gy irradiation using the RS‐2000‐PRO‐225 system (Rad Source). Twenty‐four hours later, 1 × 10^5^ HSPCs were transplanted into recipient mice via the tail vein [14]. In this model, three types of donor‐derived cells were included: Male C57BL/6J^*egfp*^ HSPCs were used for model construction and verification by EGFP^+^ cells and Y chromosome [14]. Female C57BL/6J^*egfp*^ HSPCs were employed to evaluate the extent of haematopoietic recovery and to mitigate the impact of an unmatched sex. Female C57BL/6J^*wt*^ HSPCs were utilised for tests using flow cytometry and to prevent channel occupancy of GFP. In the mobilisation‐based transplantation model, recipients were intraperitoneally administered AMD3100 (5 mg/kg; Selleck), a CXCR4 antagonist with the therapeutic potential of stem‐cell mobilisation, followed by granulocyte colony‐stimulating factor (G‐CSF; 200 μg/kg/day; Novoprotein) for 5 days [15, 16]. The transplantation method is identical to that used in TBI‐based HSCT. In the resveratrol experiments, 2.5 mg/kg/day of resveratrol (Selleck) was administered orally (in water) for a period of seven consecutive days prior to transplantation and continued until 1 month after transplantation.

### 2.4 Flow Cytometry

Bone marrow cells were isolated as previously described. Peripheral blood mononuclear cells (PBMCs) were isolated using Ficoll–Hypaque (Sigma‐Aldrich), and splenocytes were isolated using the GentleMACS Tissue Dissociator (Miltenyi). All cells were filtered using a 0.70 μm sterile filter and subsequently lysed using the lysis buffer. The proportion of EGFP‐expressing cells was detected using a FACSCanto SORP cytometer (BD Biosciences, San Jose, CA, USA) and analysed using the FlowJo software version 10 (BD Biosciences).

### 2.5 CyTOF and Data Analysis

Bone marrow and spleen cells were stained according to the Maxpar cell surface staining protocol (Fluidigm PN 400276 A1 Protocol C, version 1.1) and diluted to 1.1 × 10^6^ cells/mL for detection using a Helios system (Standard BioTools, South San Francisco, CA, USA). Each sample yielded Flow Cytometry Standard (FCS) files comprising 100,000 events. The data were analysed using the following pipeline: normalisation by *Normalizer* [17]; debarcoding by *premessa* [18]; removal of beads, filtering of dead and dimmer cells using the Cytobank [19]; and identification of subsets and performance of differential analysis using *cytofWorkflow* [20, 21, 22] in R version 4.2.0. The complete list of antibodies and reagents utilised for CyTOF is presented in Table S1.

### 2.6 RNA Sequencing and Data Analysis

Total RNA was extracted using TRIzol (Invitrogen) [23] and evaluated using an Agilent 2100 Bioanalyzer (Agilent Technologies, Palo Alto, CA, USA). The resulting data indicated that RNA concentrations exceeding 100 ng/μL, total RNA quantities greater than 2 μg, and integrity values exceeding nine were deemed suitable for library sequencing. Library preparation and sequencing were conducted by Novogene Co. (Beijing, China). The generated data were subjected to two‐way differential expression analysis using the DEseq2 package. Genes with a corrected *p*‐value ≤ 0.05 and |Log_2_FoldChange (Log_2_FC)| ≥ 1 were identified as the differentially expressed genes (DEGs). Subsequently, the DEGs were subjected to functional enrichment analysis using Metascape (https://metascape.org).

### 2.7 scRNA Sequencing Data Analysis

The single‐cell RNA transcriptomic sequencing (scRNA‐seq) data from GSE228645 [24] was obtained from the Gene Expression Omnibus (GEO) database (https://www.ncbi.nlm.nih.gov/geo/). The datasets were filtered to remove low‐quality cells with a mitochondrial content of > 5% and a ribosomal content of > 40%. Subsequently, all mitochondrial and ribosomal genes were removed from the datasets. The final, clean data were analysed and visualised using *Scanpy* version 1.9.1 in Python version 3.9.

### 2.8 Cytometric Bead Array (CBA) Assay

On the 30th day post‐transplantation, 100 μL of whole blood was collected from anaesthetised mice in advance and stored at −80°C for future use. Two months post‐HSCT, the serums were divided into two groups based on the survival of HSCT mice: live and dead. These samples were then mixed separately to form the mixture. The expression levels of major inflammatory factors, including IL‐6, IL‐10, TNF‐α, IFN‐γ and MCP‐1, were subsequently detected using the CBA Mouse Inflammation Kit (BD Biosciences #552364) in accordance with the instructions provided.

### 2.9 Enzyme‐Linked Immunosorbent Assay

Enzyme‐linked immunosorbent assay (ELISA) kits from RayBiotech were utilised to quantify the levels of IL‐6, IL‐17, TNF‐α, MCP‐1 and IFN‐γ in serum samples, in accordance with the instructions provided by the manufacturer. The absorbance of the samples at 450 nm was measured using a Synergy HTX multi‐mode reader (BioTek, Winooski, VT, USA), and the concentrations were determined through the use of standard curves.

### 2.10 Hepatorenal Function Tests

A total of 200 μL of serum was utilised for the assessment of hepatorenal function via the Automatic Biochemical Analyser (MS‐480; MedicalSystem, Suzhou, China). The analysis encompassed alanine aminotransferase (ALT), aspartate transferase (AST), alkaline phosphatase (ALP), albumin (ALB), total protein (TP), creatinine (Crea), serum urea (Urea), uric acid (UA) and triglycerides (TG).

### 2.11 DNA Fluorescence In Situ Hybridization

The Y chromosome‐specific DNA probes and regents were procured from Guangzhou EXON Biological Technology (FOCOFISH, Guangzhou, China). Bone marrow smears from ctrl and HSCT mice were prepared by adding bone marrow cells dropwise to pre‐cooled slides. The smears were then baked at 65°C for 2 h and hybridised in accordance with the manufacturer's protocol. Images were captured using an inverted microscope (ECLIPSE Ti2; Nikon, Minato‐ku, Tokyo).

### 2.12 Mouse Colony Forming Unit (CFU) Assay

HSPCs were isolated from mice 30 days post‐transplantation, diluted to a concentration of 1 × 10^5^/mL in 150 μL of IMDM media, and transferred into 1.5 mL of MethoCult media (StemCell Technologies). Subsequently, 1.1 mL of the mixture was transferred into a 35‐mm SmartDish (StemCell Technologies) with a 16G flat‐tip needle. Subsequently, the dish was incubated for seven continuous days under 5% CO₂ and 99% humidity. Thereafter, the number of colonies of the burst‐forming unit‐erythroid (BFU‐E), granulocyte and/or macrophage progenitor cells (CFU‐GM) and multi‐potential progenitor cells (CFU‐GEMM) were enumerated using the BioTek Lionheart FX Automated Microscope (Agilent).

### 2.13 Giemsa Staining of Bone Marrow Smears

Bone marrow was obtained by subjecting the bone fragments to an accelerated centrifugation pulse at 1,600 g for three cycles. Subsequently, the cell precipitates were resuspended in 20 μL of PBS, and 5 μL of the resulting solution was added dropwise to the slide. Subsequently, the bone marrow was meticulously dislodged with the assistance of an additional slide. Thereafter, the smears were fixed in acetone for 1 min and then permitted to dry naturally. The samples were stained in modified Giemsa staining solution (Beyotime) for 45 min, rinsed with running water, and then photographed under a microscope.

### 2.14 Peripheral Blood Haematology and Complete Blood Count

The blood was collected from the anaesthetised mice and added to EDTA anticoagulation tubes. The preparation of blood smears and Giemsa staining was conducted in accordance with the established methodology for bone marrow smears. The remaining whole blood samples were transported to a veterinary haematology analyser (Mindray Animal Medical; BC‐5000vet) for complete blood count analysis.

### 2.15 Statistical Analysis

The data were presented as means ± standard error and evaluated using the Shapiro–Wilk test to assess normality and Levene's test to assess variance in homogeneity. The independent Student's *t*‐test was employed for two‐group comparisons, and the one‐way ANOVA for multiple‐group comparisons, provided that both assumptions were satisfied. Otherwise, the Mann–Whitney *U* test or Kruskal–Wallis test was utilised. Survival was analysed using the Kaplan–Meier method and compared with the log‐rank test. All statistical analyses were conducted and visualised using Prism version 9.0 (GraphPad). All tests were two‐tailed, and a *p*‐value of < 0.05 was considered statistically significant.

## 3 Results

### 3.1 HSCT Results in Heightened Inflammatory Response of Non‐GvHD Origin Within 30 Days Following Allo‐HSCT in Patients

**FIGURE 1:**
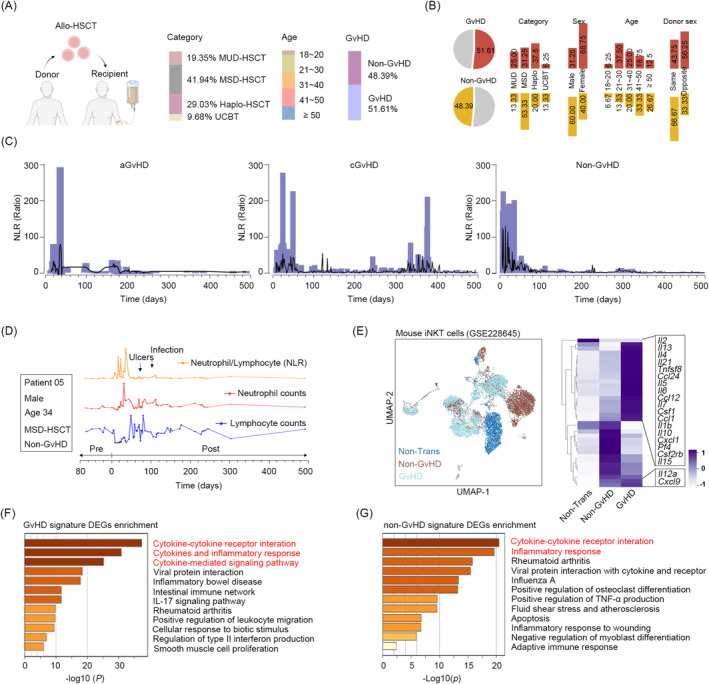
HSCT results in heightened inflammation of non‐GvHD origin within 30 days following allo‐HSCT in patients and mice. (A) Baseline characteristic of 31 SAA patients, including transplant type, GvHD and age. (B) The clinical characteristics of patients according to their GvHD condition. (C) Neutrophil/lymphocyte ratios (NLR) in patients with aGvHD (left), cGvHD (middle), and non‐GvHD (right) over a 500‐day period following allo‐HSCT. (D) Peripheral blood lymphocyte (blue line) and neutrophil (red line) counts, as well as NLR (orange line) of patient 05. (E) UMAP plot (left) of GSE228645 and heatmap (right) demonstrating the mRNA levels of genes involved in the inflammatory response. (F, G) Metascape analysis showing the enriched pathways that are enriched for feature genes in mice with and without GvHD.

In order to investigate the levels of inflammation following HSCT, we collected peripheral blood data from 31 patients with SAA who underwent allo‐HSCT. Among the patients, 19.35% underwent matched unrelated donor HSCT (MUD‐HSCT), 41.94% underwent MSD‐HSCT, while 29.03% and 9.68% received haplo‐HSCT and umbilical cord blood transplantation (UCBT), respectively (Figure 1A). It was observed that 51.61% of the patients experienced either acute or chronic GvHD, of which cGvHD accounted for 45.16%, aGvHD accounted for 18.18% and all of them underwent haplo‐HSCT (Figure 1B). We thus analysed the neutrophil/lymphocyte ratios (NLR), a promising biomarker of immune dysfunction and systemic inflammation [25, 26], in aGvHD, cGvHD and non‐GvHD patients, separately. The results demonstrated that aGvHD exhibited a notable NLR surge within 2 months post‐transplantation, while cGvHD displayed a pronounced NLR peak at 300 days post‐transplantation (Figure 1C), which aligns with the onset of classical GvHD [5, 27]. The presented evidence provides support for the feasibility of utilising NLR in HSCT. It is noteworthy that our findings indicate an observed increase in NLR levels in non‐GvHD patients during the early stages post‐HSCT, and the level is significantly higher than that caused by infection and inflammatory disorders (Figure 1D). This indicates the potential existence of unidentified sources of inflammation. To test this hypothesis, we reanalyzed the dataset presented by Zhao et al. [24], who examined invariant natural killer T (iNKT) cells isolated from the thymus of mice with (GvHD) and without GvHD (Non‐GvHD). In comparison to the non‐transplant mice, both GvHD and non‐GvHD mice demonstrated elevated levels of inflammatory factors (Figure 1E). Furthermore, the GvHD mice displayed elevated expression levels of numerous interleukin family members in comparison to the non‐GvHD mice (Figure 1E). The Metascape enrichment analysis of genes characterising GvHD and non‐GvHD revealed a hyperinflammatory response unrelated to GvHD in the early post‐HSCT period (Figure 1F,G).

### 3.2 Hyperinflammation in the Initial 30‐Day Period Leads to High‐Risk TRM in Mice

**FIGURE 2:**
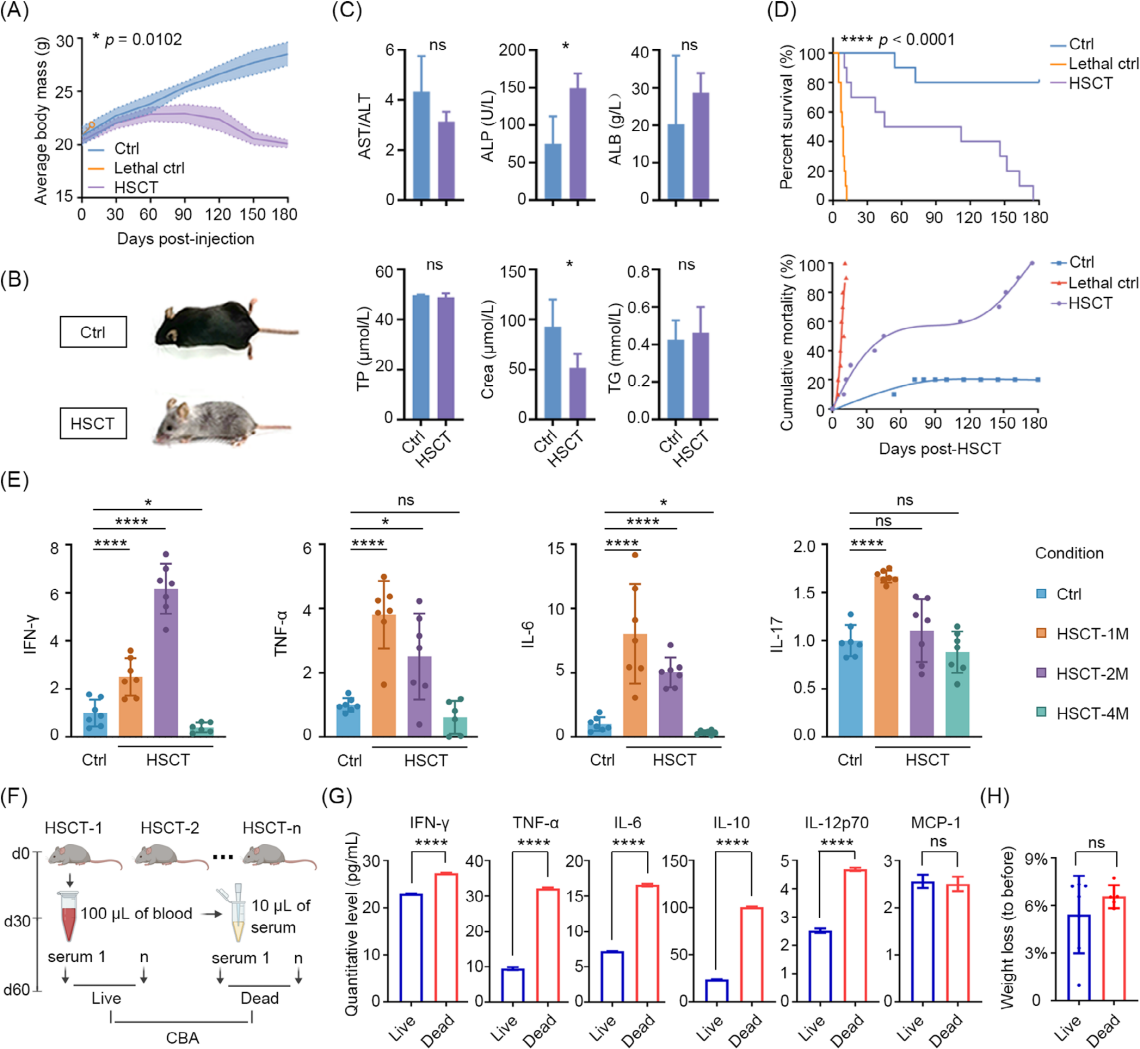
Post‐transplant inflammation in the initial 30 days leads to high‐risk TRM in mice. (A) Line chart outlining weight loss in ctrl, lethal ctrl and HSCT mice (*n* = 10). (B) Appearance of ctrl and HSCT mice at the fourth‐month post‐transplantation. (C) Quantification of the hepatorenal function indices of ctrl and HSCT mice at the above time point. (D) Percent survival (top) and cumulative mortality (bottom) in ctrl, lethal ctrl and HSCT mice (*n* = 10). (E) ELISAs showing the relative levels of serum IFN‐γ, TNF‐α, IL‐6, and IL‐17 in the different time points post‐HSCT. (F) Schematics of obtaining slight serum from each HSCT mouse at day 30 post‐transplantation, and observing those mice continuously until 2 months. (G) Cytometric bead array (CBA) assay identifying the levels of inflammatory factors present in the serum mixture obtained from mice that had survived for 2 months, in comparison to those that had not. (H) Correlation between the weight loss and survival. ns (no significance) *p* > 0.05, *p < 0.05, **p < 0.01, ***p < 0.001, ****p < 0.0001.

To further investigate the relationship between inflammation and transplantation, we established a syngeneic transplantation model in mice to eliminate the effects of GvHD by transplanting HSPCs into TBI‐treated recipients, including untreated mice (control, ctrl) and irradiated mice without transplantation (lethal control, lethal ctrl) (Figure S1A). The level of donor‐derived cells was evaluated through Y‐chromosome probe (ChrY) (*p* < 0.0001), mRNA level of *Sry* (*p* = 0.020) and EGFP^+^ cells (*p* < 0.0001) (Figure S1B–D) in recipient mice at the fourth month post‐transplantation. No difference was observed in haematopoietic stem cell function in post‐transplantation mice (Figure S1E). Although HSCT achieved haematopoietic reconstitution, our observation showed that HSCT mice showed significant weight loss (Figure 2A) and visible unhealthy changes in fur texture and body posture (Figure 2B). Meanwhile, there was a marked elevation in ALP (*p* = 0.030) and a decline in serum creatinine (Crea) (*p* = 0.026) in the HSCT mice (Figure 2C).

The results of the transplantation experiments indicated that all mice in the lethal control group succumbed to the effects of radiation exposure. Accordingly, the 14‐day survival was deemed an indicator of the efficacy of the haematopoietic stem cell transplantation. It is noteworthy that among mice that underwent HSCT and survived beyond the first 14 days, 50% died within 2 months, while the remaining 10% and 40% died within 2–4 and 4–6 months, respectively (Figure 2D). This indicates the existence of a period of elevated risk for TRM during the initial 2 months following transplantation. Subsequently, we investigated the levels of inflammatory factors associated with the transplantation process [7, 8], including IL‐6, IL‐17, TNF‐α and IFN‐γ, in serum samples obtained from HSCT and age‐matched control mice at the first, second and fourth month following transplantation. The results demonstrated a statistically significant increase in the levels of nearly all measured factors in HSCT mice during the initial 2 months. It is noteworthy that these factors reached their highest levels in the first month, with the exception of IFN‐γ, and exhibited decreased levels of specific factors, including IFN‐γ and IL‐6, by the fourth month (Figure 2E).

To ascertain the potential correlation between inflammation and TRM, we collected 100 μL of blood from each HSCT mouse via the angular vein and isolated serum. The HSCT mice were then observed for a period of 2 months, after which their serum samples were grouped according to survival outcomes and subjected to a CBA assay to quantify the levels of inflammatory factors (Figure 2F). The results demonstrated that the levels of IFN‐γ, TNF‐α, IL‐6, IL‐10 and IL‐12p70 were significantly lower in the surviving mice than in the deceased mice, with the exception of MCP‐1, which exhibited no difference (Figure 2G). However, no correlation was discerned between weight loss and survival (Figure 2H).

### 3.3 Hyperinflammation Results From Transplant Practice Except Irradiation and Mobilisation

**FIGURE 3:**
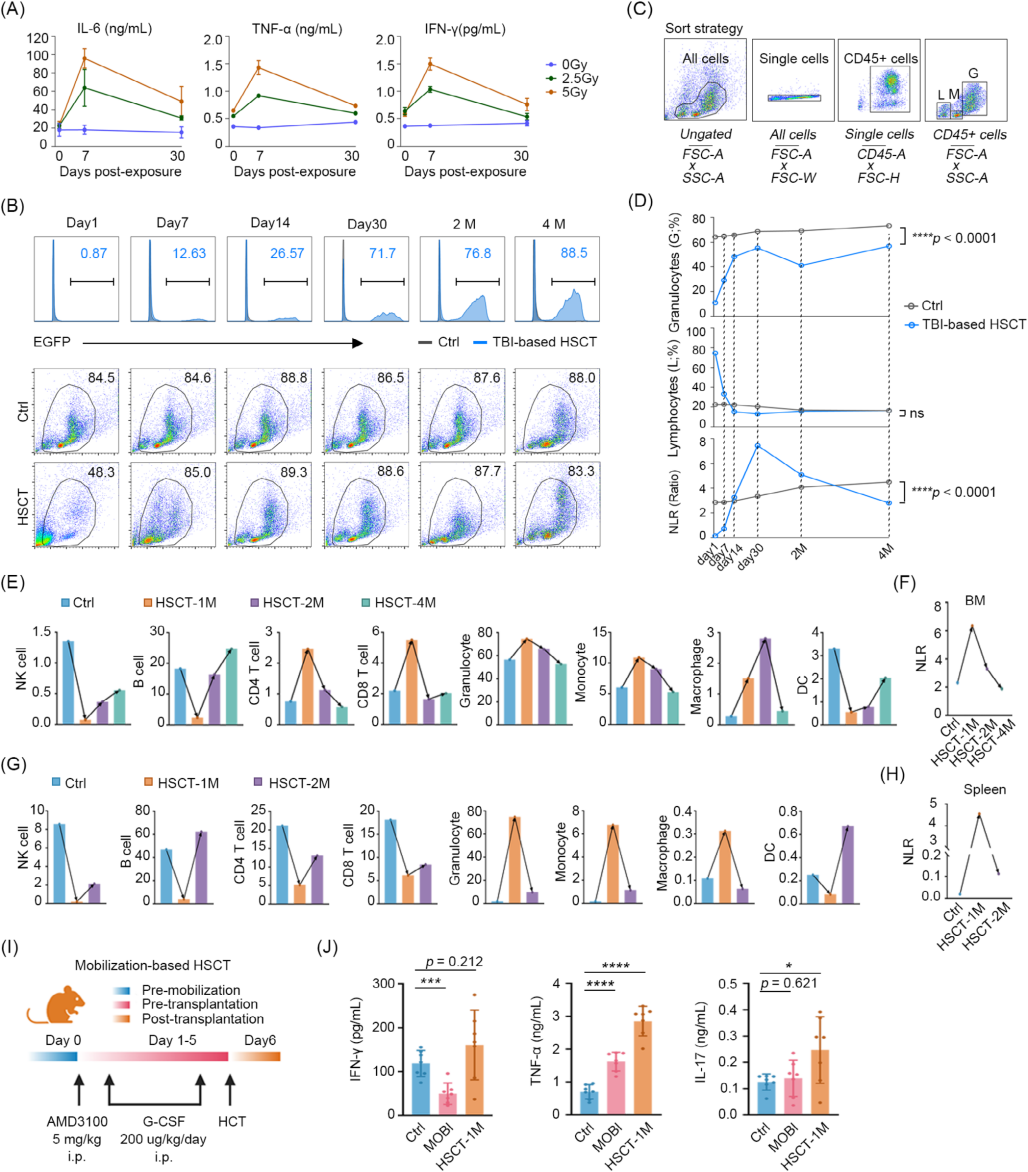
Hyperinflammation results from transplant practice except irradiation and mobilisation. (A) Levels of IL‐6, TNF‐α and IFN‐γ induced by non‐lethal doses of radiation. (B) The proportion of EGFP^+^ cells (top) and percentage of efficient cells within the bone marrow (bottom) on day 1, day 7, day 14, day 30, 2‐ and 4‐month post‐HSCT mice. Strategy of distinguishing lymphocyte‐, monocyte‐ and granulocyte‐like cells in reconstituted bone marrow (C) and the post‐transplantation trends (D). NLR values calculated by granulocyte−/lymphocyte‐like cells. CyTOF revealing the cell populations and equivalent NLR values in bone marrow (E, F) and spleen cells (G, H) from day 30, 2‐ and 4‐month ctrl and HSCT mice. (I) Schematic design for mobilisation‐based transplantation using AMD3100 and G‐CSF. (J) Levels of IFN‐γ, TNF‐α, and IL‐17 in ctrl, mobilisation alone, and mobilisation‐based HSCT mice. ns (no significance) *p* > 0.05, *p < 0.05, **p < 0.01, ***p < 0.001, ****p < 0.0001.

In order to identify the underlying causes of inflammation, we evaluated two potential sources of inflammation in the HSCT process: irradiation and hemopoietic reconstitution, respectively. Firstly, mice were treated with non‐lethal doses of 2.5Gy and 5Gy irradiation, and serum inflammation levels were examined at days 0, 7 and 30. The findings indicated that the level of inflammation was elevated in the irradiated mice in comparison to the untreated mice, and that this elevation was associated with higher doses. The highest levels of inflammation were observed on day 7 among the examined time points. Notwithstanding the persistence of elevated levels in comparison to the unirradiated group at day 30, the contribution to systemic inflammation was significantly diminished (Figure 3A). In the HSCT process, the proportion of donor‐derived cells exhibited a notable increase, from 0.87% to 88.5%. As evidenced by the presence of effective cells in the bone marrow, the 30‐day period following transplantation represented the most active phase of haematopoiesis, followed by a gradual increase (Figure 3B). By calculating the NLR of these effective cells (Figure 3C), we observed a notable increase during the 30‐day post‐transplantation period, reaching a maximum at day 30. Thereafter, NLR values continually decreased and reached levels comparable to the control at approximately 3 months post‐transplantation (Figure 3D).

Given that the haematopoietic inductive microenvironment can be modified by immune cells, cytokines and chemokines, which in turn regulate the development of haematopoietic lineages [28]. To examine the function of haematopoiesis in the context of inflammation, we analysed the immune profiles of bone marrow and spleen cells collected from mice at 1, 2 and 4 months post‐HSCT using CyTOF (Figure S2A–D). The results demonstrated the notable alteration in the composition of immune subsets during the initial month following HSCT in both bone marrow and spleen. By the fourth month, the haematopoietic lineage had almost recovered (Figure 3E,G). However, NK cells and dendritic cells (DCs) exhibited a gradual recovery to their previous levels in terms of numbers (Figure S2E–F). In accordance with the ELISA results, the NLR increased continuously in the 30 days following transplantation (Figure 3F,H). This indicates that the imbalance in granulocytes and lymphocytes, resulting in a myeloid bias, may contribute to inflammation.

To further exclude high doses of irradiation‐induced senescence‐associated secretory phenotype (SASP) [29, 30] and radioactive damage to the parenchymal organs, including the liver, kidneys and intestine [31, 32, 33], we performed mobilisation‐based HSCT in mice (Figure 3I). Mobilisation‐based transplantation is a common clinical approach in autologous HSCT, which employs ‘de‐adhesion’ agents to mobilise HSCs migrating from the bone marrow to the periphery [33]. The results indicated that mobilisation resulted in a minimal elevation in TNF‐α, whereas the levels of TNF‐α and IL‐17 were markedly elevated following transplantation (Figure 3J). It is noteworthy that the number of donor‐derived cells was markedly reduced after 30 days in the context of mobilisation‐based transplantation (Figure S3A). This type of transplantation is unlikely to cause injury to the live kidney and colon (Figure S3B–D), which limits the suitability of this model for studies observing TRM and long‐term effects.

### 3.4 Low‐Inflammatory Mice Demonstrate Downregulation of NOD‐Like Receptor Signalling Pathway Activity

**FIGURE 4:**
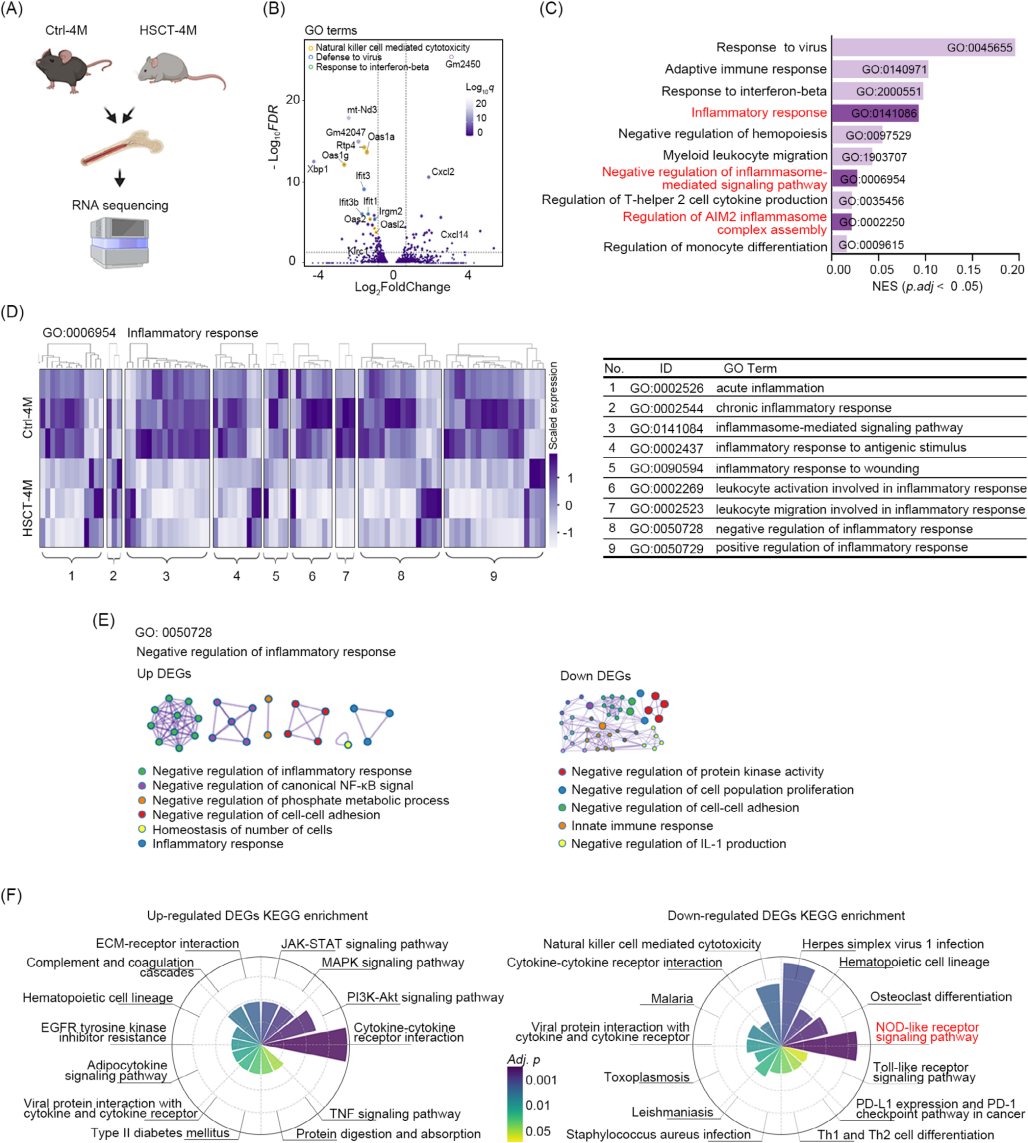
Low‐inflammatory mice demonstrate downregulation of NOD‐like receptor signalling pathway activity. (A) Schematic design for RNA sequencing of bone marrow cells from ctrl and the fourth month HSCT mice (*n* = 3). (B) GO term volcano plot showing genes with decreased expression in GO pathways such as NK cell‐mediated cytotoxicity, defence against virus, and response to interferon‐beta. (C) Top 12 GO enrichment pathways of all DEGs (adjusted *p* < 0.05). (D) Expression heatmap of primary DEGs (*p* < 0.05) in the inflammatory response gene set (GO term: 0006954). (E) Protein–protein interaction network showing the Metascape results of upregulated (top) and downregulated (bottom) genes in GO term: 022834, a negative subset of inflammation regulation. (F) Polar plots showing KEGG enrichment analysis results of the upregulated (left) and downregulated (right) pathways.

To further elucidate the potential mechanisms underlying anti‐inflammatory and TRM processes, we conducted an in‐depth analysis of the RNA‐seq data from the bone marrow of control and 4‐month low‐inflammatory HSCT mice (Figure 4A). This analysis led to the identification of DEGs (Figure 4B). The gene ontology (GO) enrichment analysis revealed that DEGs were enriched in inflammasome and inflammatory response‐mediated signalling pathways (Figure 4C). These pathways constitute branches of the inflammatory response pathway (GO term: 0006954). Therefore, we proceeded to investigate the expression levels of genes involved in the GO term 0006954. The results demonstrated that the majority of the genes exhibited decreased expression levels, while only a minority demonstrated increased expression levels in the HSCT mice (Figure 4D). The Metascape enrichment analysis of the up‐ and down‐regulated genes, for instance, in the negative regulation of the inflammatory response pathway (GO term: 0050728), revealed that the upregulated genes were involved in the inhibition of inflammation, while the downregulated genes were primarily associated with innate immunity and cellular processes (Figure 4E). Consequently, the HSCT mice exhibited a dual state of mitigated inflammation and suppressed immune system activity. Subsequently, the KEGG enrichment analysis yielded analogous results, with the JAK–STAT, PI3K/Akt and MAPK pathways being upregulated and the NOD‐like receptor pathway and toll‐like receptors being downregulated in the HSCT mice (Figure 4F).

### 3.5 Administration of the Resveratrol Reduces Inflammation and TRM by Modulating Haematopoiesis

**FIGURE 5:**
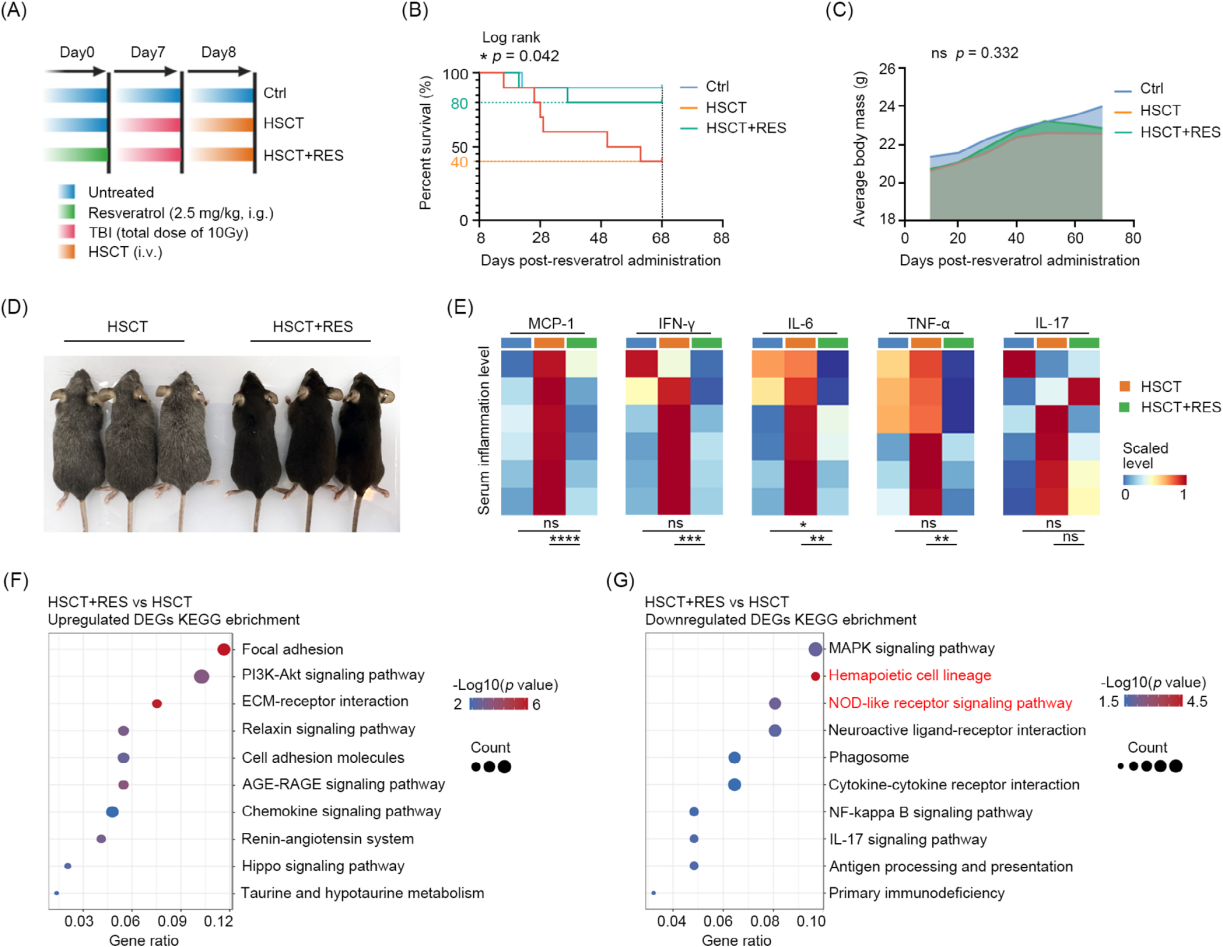
Administration of the resveratrol reduces inflammation and transplant‐related mortality by modulating haematopoiesis. (A) Schematic design for resveratrol administration in HSCT mice. Survival (B) and weight loss (C) in ctrl, HSCT, and HSCT‐RES groups (*n* = 10). (D) Appearance of HSCT and HSCT‐RES mice at the day 30 post‐transplantation. (E) ELISAs demonstrating the levels of MCP‐1, IFN‐γ, IL‐6, IL‐17, and TNF‐α in the indicated groups. KEGG enrichment results for the upregulated (F) and downregulated (G) pathways in HSCT‐RES mice compared to normal HSCT mice. ns (no significance) *p* > 0.05, *p < 0.05, **p < 0.01, ***p < 0.001, ****p < 0.0001.

Given the established correlation between 30‐day inflammation and TRM, we sought to investigate whether anti‐inflammatory therapy might confer beneficial effects in HSCT. Resveratrol, an inhibitor of the NOD‐like receptor with broad‐spectrum anti‐inflammatory effects in various inflammatory diseases [34], was administered to the HSCT mice at a dose of 2.5 mg/kg/day continuously from 7 days before transplantation until 30 days after transplantation (Figure 5A). The results demonstrated that, in comparison to the HSCT mice that were not treated with resveratrol, those that were treated with resveratrol exhibited enhanced 2‐month survival (80% vs. 40%; *p* = 0.042) (Figure 5B). However, this was not accompanied by an improvement in weight loss (Figure 5C). It is noteworthy that resveratrol demonstrated additional protective effects, including notable benefits in the context of fair damage (Figure 5D) and reductions in levels of pro‐inflammatory factors such as IL‐6, TNF‐α, IFN‐γ, IL‐17 and MCP‐1 (Figure 5E). Moreover, resveratrol exhibited efficacy in alleviating hepatocellular edema, vacuolization and steatosis of hepatocytes (indicated by black arrows), as well as the beneficial decrease in ALT levels in TBI‐based HSCT mice (Figure S4A,B). Additionally, it mitigated glomerular atrophy and glomerular cystic lumen enlargement, and reduced levels of urea and UA post‐transplantation (Figure S4C–D). The bone marrow RNA‐seq data from HSCT‐RES and HSCT mice at 30 days post‐transplantation, indicated that resveratrol downregulates the haematopoietic cell lineage (mmu04640) and NOD‐like receptor signalling pathways (mmu04621), and the involved genes included *Il1r2*, *Cd14*, *H2‐Eb2*, *Il9r*, *Cd8b1* and *Cd59* (Figure 5F,G). Concurrently, resveratrol enhanced the colonisation‐associated pathways of HSCs, including the cell adhesion molecule pathway (mmu04510) and the hippo signalling pathway (mmu04390), and increased the prevalence of red lineages, such as red blood cell (RBC), haemoglobin (HGB), platelets (PLT) and mean platelet volume (MVP) (Figure S4E). The results indicate that the observed haematopoietic deceleration is likely due to transient suppression of inflammatory mediators, rather than a disruption in HSC functionality.

### 3.6 Delayed Reconstitution of Hemopoiesis by Resveratrol Can Be Restored During a Brief Period

**FIGURE 6:**
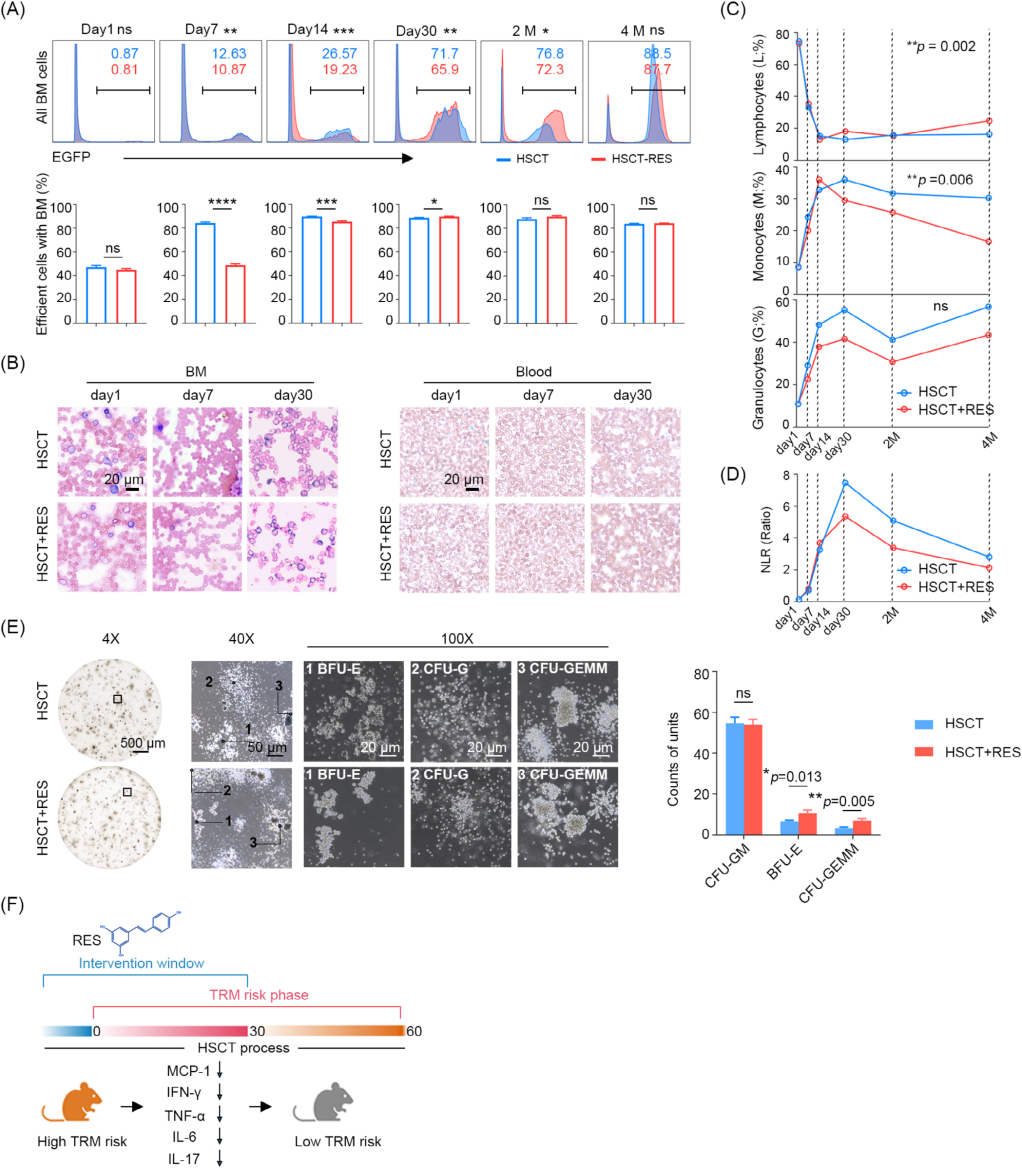
Delayed reconstitution of hemopoiesis by resveratrol can be restored during a brief period. (A) Flow cytometry showing the proportion of EGFP^+^ cells (top) and percentage of efficient cells on day 1, day 7, day 14, day 30, 2‐ and 4‐month post‐HSCT in HSCT and HSCT‐RES mice. (B) Giemsa stained smear showing the myelogram (left) and hemogram (right) on day 1, day 7 and day 30 in the mice mentioned above. (C) Lymphocyte‐, monocyte‐ and granulocyte‐like cells in HSCT and HSCT‐RES mice. (D) The post‐transplantation trends of NLR. (E) CFU assay of HSPCs from the bone marrow of HSCT and HSCT‐RES mice. (F) Schematic illustrating the mechanism of resveratrol in reduce the risk of TRM. ns (no significance) *p* > 0.05, *p < 0.05, **p < 0.01, ***p < 0.001, ****p < 0.0001.

Given the delayed effect of resveratrol on haematopoiesis, it is necessary to investigate the potential impact of resveratrol on haematopoietic recovery. The percentages of bone marrow EGFP^+^ cells were first compared in TBI‐based HSCT (HSCT) and TBI‐based HSCT with oral resveratrol (HSCT‐RES) mice on days 1, 7, 14, 30, and at the second and the fourth‐month post‐transplantation. The results demonstrated that, in comparison to normal HSCT, mice that received oral resveratrol exhibited a lower percentage of EGFP^+^ cells on days 7, 14, 30 post‐HSCT (Figure 6A). In mice that underwent HSCT, the bone marrow exhibited a distinct presence of granulocytes and monocytes on day 1 and apparent lymphocytes on day 7, with discernible lymphocyte‐, monocyte‐ and granulocyte‐like populations until day 14. However, we could not identify the lymphocyte clusters on day 7 or distinct clusters on day 14 in HSCT‐RES mice. Interestingly, the results indicated a promising trend: HSCT‐RES mice exhibited a notable reduction in the percentage of monocyte‐ and granulocyte‐like clusters by the 30th day post‐transplantation in comparison to the untreated HSCT mice. This is verified by an increase in the number of naïve cells in the myelogram, indicating a delay in reconstitution (Figure 6B). However, 1 month after cessation of resveratrol intake (i.e., the second month after transplantation), the effective cells in the bone marrow of HSCT‐RES mice returned to normal levels. By the third month after cessation (i.e., the fourth month after transplantation), there was no longer a statistically significant difference in the chimeric cells in the bone marrow between the two groups, indicating that the delayed haematopoietic effect of resveratrol can be restored within a relatively short period of time following cessation of intake (Figure 6A,B). Interestingly, results of the lymphocyte‐, monocyte‐ and granulocyte‐like clusters indicated a promising trend: HSCT‐RES mice exhibited a notable reduction in the percentage of monocyte‐ and granulocyte‐like clusters by the 30th‐day post‐transplantation in comparison to the untreated HSCT mice (Figure 6C). The results of the NLR demonstrated that the HSCT‐RES mice exhibited a lower level of inflammation than the HSCT mice at the corresponding time points (Figure 6D).

The function of haematopoietic stem cells has an impact on the recovery of haematopoiesis [35, 36, 37, 38]. To test whether resveratrol impacts the differentiation capacity of HSCs, we isolated the Haematopoietic Stem and Progenitor Cells (HSPCs) from Haematopoietic Stem Cell Transplantation (HSCT) and HSCT with oral resveratrol mice on day 30 post‐transplantation, and performed Colony‐Forming Unit (CFU) assays. The results demonstrated that counts of GEMM and BFU‐E were found to be enhanced, while the number of CFU‐GM remained unaltered (Figure 6E), indicating that administration of resveratrol had no impact on the differentiation capacity of haematopoietic stem cells in normal mice. The results of this study indicate that the myeloid bias originated from haematopoietic reconstitution may contribute to the elevated levels of inflammation and high risk of TRM. It is hypothesized that resveratrol exerts its effects by reducing myeloid bias, thereby regulating post‐transplantation inflammation levels and TRM risk (Figure 6F).

## 4 Discussion

The present study is a comprehensive investigation of post‐transplantation alterations of inflammatory mediators and the correlation between inflammation level and TRM. The 30‐day period following transplantation is distinguished by the most elevated inflammatory response, which increases the risk of TRM. This provides a potential avenue for the exploitation of anti‐inflammatory treatments. The inflammation is primarily caused by myeloid bias during haematopoietic reconstitution and, to a lesser extent, irradiation‐induced injury. The administration of anti‐inflammatory agents can prove beneficial in reducing TRM and improving post‐transplant survival. The findings suggested the possibility of a promising approach to controlling inflammation in the early stages of transplantation.

GvHD has been previously identified as a source of post‐transplantation inflammation [5, 6]. To evaluate the degree of inflammation in patients undergoing HSCT, we utilised an index, the NLR. The NLR is a recognised indicator that can represent the systemic inflammation in many disease studies, including those of tumours and infections [39, 40]. Recently, the NLR has been reported to predict GvHD [26, 41]. Our analysis of NLR in patients with cGvHD also demonstrated a notable increase in NLR values coinciding with the onset of the GvHD cycle. These observations lend support to the use of NLR for the assessment of post‐transplant inflammation. It is noteworthy that our findings indicated a peak of NLR in patients who did not develop GvHD, which suggests the presence of unidentified sources of inflammation in the HSCT process. To study the inflammatory causes while eliminating the influence of GvHD, we developed a syngeneic transplant model in mice. The model revealed that the peak levels of inflammation occur on day 30 post‐transplantation. Furthermore, elevated levels of inflammatory factors, including IL‐6, IL‐17, TNF‐α, IFN‐γ and IL‐12p70, have been linked to an increased risk of TRM. A comparative analysis of the potential sources of inflammation in TBI‐based HSCT revealed that irradiation‐induced inflammation initially exhibited a higher level, which subsequently diminished. In contrast, myeloid bias‐induced inflammation demonstrated a consistent trend with systemic inflammation, as observed. As evidenced by RNA‐seq of mice with low inflammatory responses, which exhibited a shared down‐regulated NOD‐like receptor signalling pathway. The NOD‐like and toll‐like receptors have been demonstrated to promote the innate immune response by detecting pathogen‐ and damage‐associated molecular patterns generated during bone marrow ablation. This, in turn, leads to the release of pro‐inflammatory factors [42, 43]. Consequently, the administration of NOD‐like receptor inhibitor resveratrol has been shown to result in a significant improvement in HSCT. The RNA‐seq data of HSCT‐RES and HSCT mice revealed that resveratrol markedly downregulated the haematopoietic cell lineage (mmu04640) and the NOD‐like receptor signalling pathways (mmu04621). It seems reasonable to posit that the use of resveratrol reduced the release of NOD‐related inflammatory factors, and subsequently downregulated the genes involved in immune cells (*Cd14*, *Cd8b1*), thereby delaying haematopoiesis. This was demonstrated by a reduction in the proportion of granulocyte‐like cells and a decrease in the NLR value. The regulation of granulocyte and platelet implantation may result in enhanced outcomes, whereas accelerated haematopoietic recovery may potentially result in adverse effects, including GvHD [44]. These findings indicate that resveratrol may regulate “reconstruction‐induced inflammation” by effectively slowing granulocyte implantation. Fortunately, our long‐term assessment of resveratrol's impact on HSC functionality revealed that resveratrol did not impair HSC function. Instead, it demonstrated the potential to enhance stemness and colonisation.

Furthermore, additional benefits of resveratrol were identified, including the alleviation of liver and kidney damage, improvement of hair damage and other potential applications that may warrant further investigation.

‘Reconstitution‐induced inflammation’ refers to the phenomenon of myeloid bias arising from haematopoietic imbalance. This phenomenon has been frequently disregarded in the context of clinical transplantation. The objective of this study is to test the hypothesis that the use of adjuvant anti‐inflammatory therapy to reduce reconstitution‐induced inflammation could potentially mitigate transplant‐related adverse effects, facilitate HSCT colonisation and provide long‐term benefits. In some non‐myeloablative or mini‐transplant/mini‐allograft allogeneic HSCTs, this contribution may be more significant. However, as the slowing down of haematopoiesis has the potential to increase post‐transplantation risks, it is essential to exercise strict control over the dosage and timing of resveratrol administration.

Previous studies have reported limited mouse survival from lethal doses of irradiation [45]. However, in our study, no mice survived. Our findings indicate that weight loss is not statistically significantly correlated with survival and is not attenuated by resveratrol. This suggests that weight loss may be a common side effect of TBS‐based HSCT. Additionally, we intend to confirm our results in allogeneic and xenogeneic mice for further investigation.

## Author Contributions

**Xiao Zhang:** conceptualization (equal), investigation (lead), methodology (lead), resources (equal), writing – original draft (lead). **Wei Yu:** data curation (equal), investigation (equal), methodology (equal), visualization (equal), writing – review and editing (equal). **Yimeng Sun:** data curation (equal), investigation (equal), resources (equal). **Xinyu Ye:** formal analysis (equal), investigation (equal). **Yu He:** formal analysis (equal), investigation (equal). **Xin Huang:** formal analysis (equal), investigation (equal). **Fuhao Wang:** formal analysis (equal), investigation (equal). **Yi Lu:** conceptualization (equal), funding acquisition (equal), methodology (equal), resources (equal), supervision (equal), writing – review and editing (equal). **Jian Zhang:** conceptualization (equal), funding acquisition (equal), methodology (equal), project administration (equal), supervision (lead), writing – review and editing (equal).

## Ethics Statement

Animal experiments were performed according to protocols approved by the Institutional Animal Care and Use Committee at Laboratory Animal Center of Southern University of Science and Technology. Patients data collection was approved by the School of Clinical Medicine of Weifang Medical University (approval No. 2023YX133).

## Consent

All patients were informed consent to use their data for research purposes.

## Conflicts of Interest

The authors declare no conflicts of interest.

## Supporting information


Data S1.

## Data Availability

CyTOF data supporting the conclusions of this article is available in the FlowRepository (http://flowrepository.org/) with number of FR‐FCM‐Z729. Further information and requests for resources and reagents should be directed to the lead contact, Jian Zhang (zhangjian@sustech.edu.cn).
