# Supplementary material for: Anti‐Inflammatory Resveratrol Protects Mice From Early Mortality After Haematopoietic Stem Cell Transplantation
Source: J Cell Mol Med. 2025 Feb 3;29(3):e70395. doi: 10.1111/jcmm.70395 (PMC11790355; doi:10.1111/jcmm.70395)
Supplement: Supplementary file 1 — Data S1. [file JCMM-29-e70395-s001.pdf]

Supplementary Materials for

Anti-inflammatory resveratrol protects mice from early mortality after hematopoietic stem cell transplantation

Xiao Zhang *et al.*

*Corresponding author. Email: zhangjian@sustech.edu.cn

This PDF file includes:

Figures S1 to S4

Legends for figures S1 to S4

Tables S1

Captions for tables S1

Figure S1

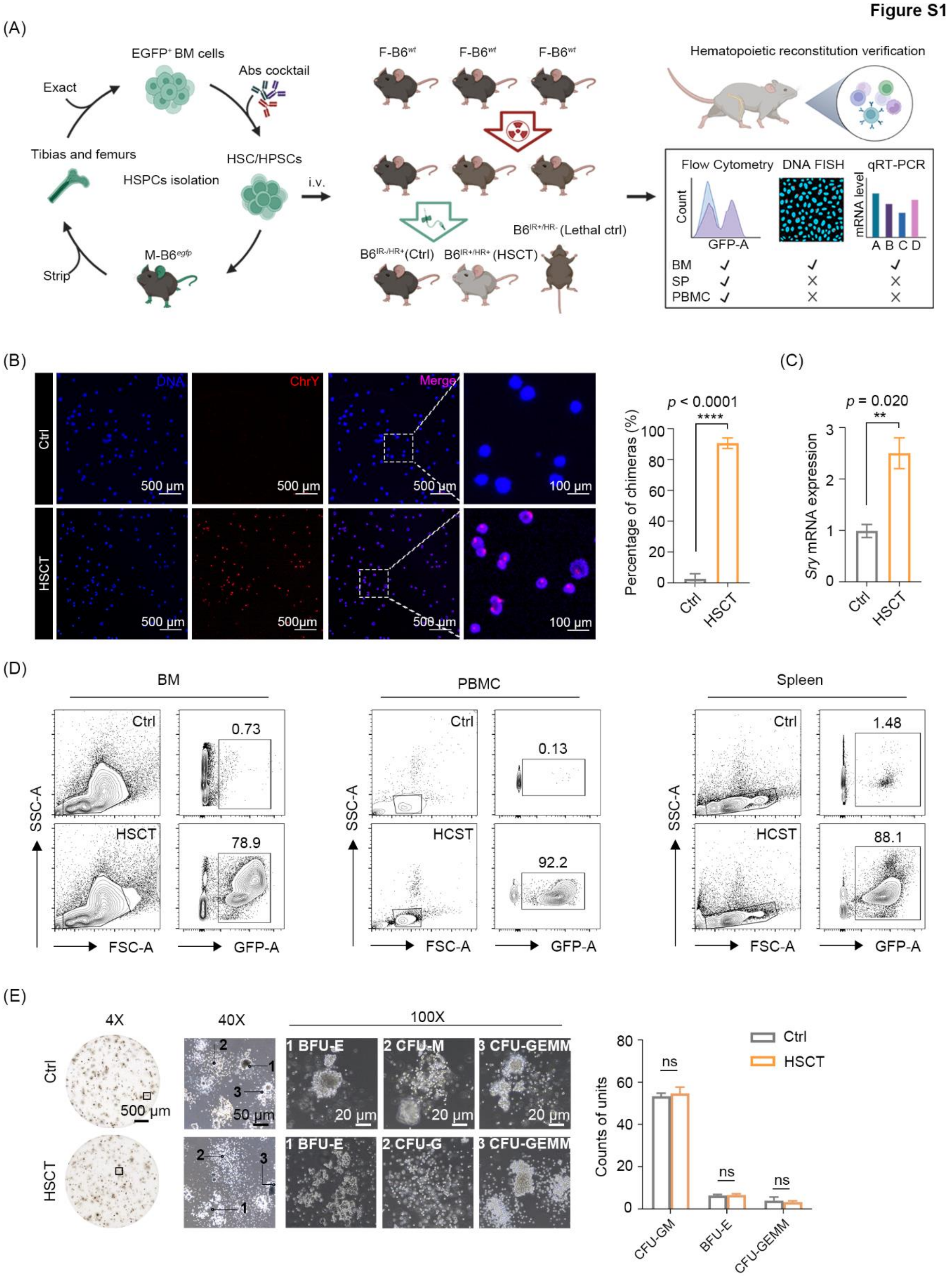
(A)
EGFP+ BM cells
Exact
Abs cocktail
Tibias and femurs
HSC/HPSCs
HSPCs isolation
i.v.
Strip
M-B6egfp
F-B6wt
B6IR-/HR+(Ctrl)
B6IR+/HR+(HSCT)
B6IR+/HR-(Lethal ctrl)
Hematopoietic reconstitution verification
Flow Cytometry
DNA FISH
qRT-PCR
Count
GFP-A
mRNA level
A B C D
BM
SP
PBMC
(B)
DNA
ChrY
Merge
Ctrl
HSCT
500 μm
100 μm
p < 0.0001
****
Percentage of chimeras (%)
(C)
p = 0.020
**
Sry mRNA expression
(D)
BM
PBMC
Spleen
Ctrl
HSCT
HCST
0.73
78.9
0.13
92.2
1.48
88.1
SSC-A
FSC-A
GFP-A
(E)
4X
40X
100X
1 BFU-E
2 CFU-M
3 CFU-GEMM
2 CFU-G
50 μm
20 μm
Ctrl
HSCT
ns
Counts of units
CFU-GM
BFU-E
CFU-GEMM

Figure S2

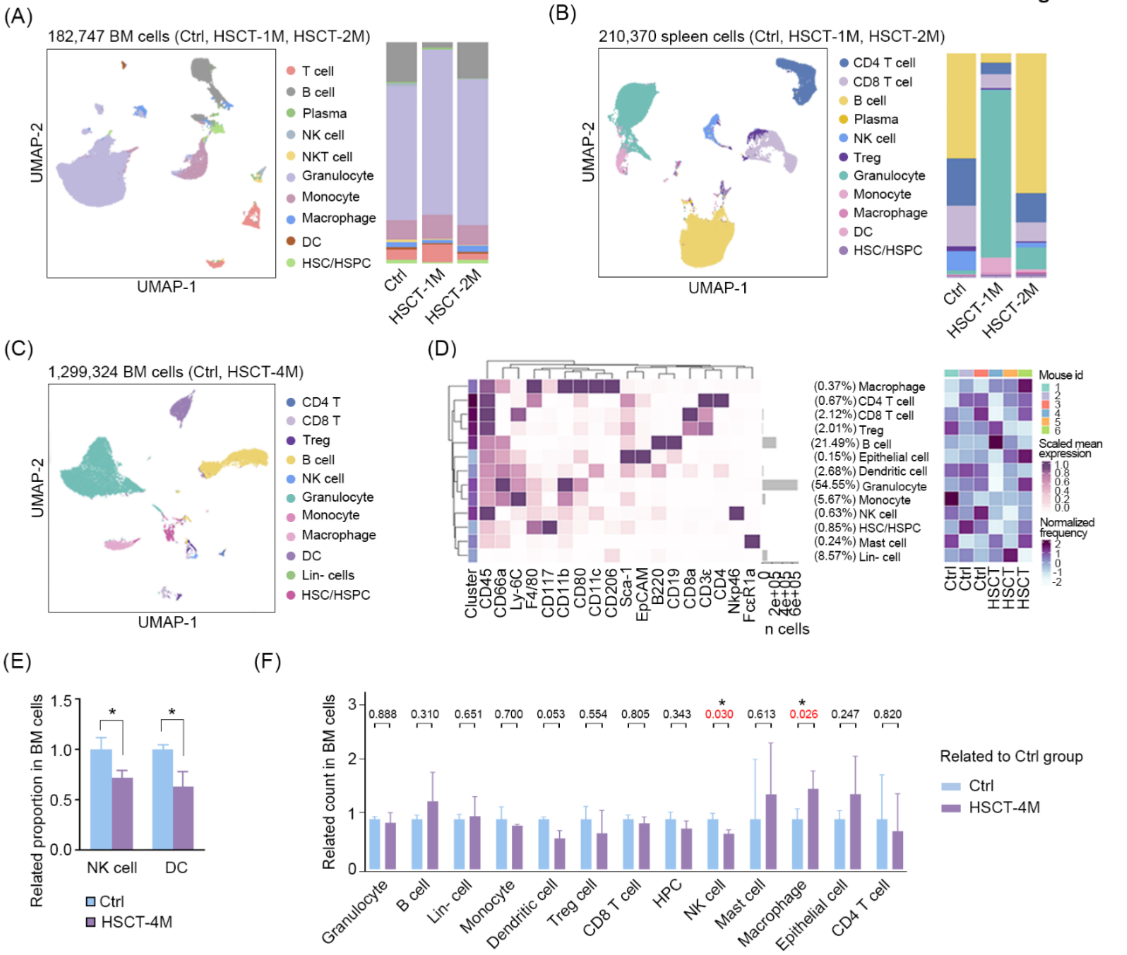
(A)
182,747 BM cells (Ctrl, HSCT-1M, HSCT-2M)
UMAP-2
UMAP-1
T cell
B cell
Plasma
NK cell
NKT cell
Granulocyte
Monocyte
Macrophage
DC
HSC/HSPC
Ctrl
HSCT-1M
HSCT-2M
(B)
210,370 spleen cells (Ctrl, HSCT-1M, HSCT-2M)
UMAP-2
UMAP-1
CD4 T cell
CD8 T cel
B cell
Plasma
NK cell
Treg
Granulocyte
Monocyte
Macrophage
DC
HSC/HSPC
Ctrl
HSCT-1M
HSCT-2M
(C)
1,299,324 BM cells (Ctrl, HSCT-4M)
UMAP-2
UMAP-1
CD4 T
CD8 T
Treg
B cell
NK cell
Granulocyte
Monocyte
Macrophage
DC
Lin- cells
HSC/HSPC
(D)
(0.37%) Macrophage
(0.67%) CD4 T cell
(2.12%) CD8 T cell
(2.01%) Treg
(21.49%) B cell
(0.15%) Epithelial cell
(2.68%) Dendritic cell
(54.55%) Granulocyte
(5.67%) Monocyte
(0.63%) NK cell
(0.85%) HSC/HSPC
(0.24%) Mast cell
(8.57%) Lin- cell
Cluster
CD45
CD66a
Ly-6C
F4/80
CD117
CD11b
CD80
CD11c
CD206
Sca-1
EpCAM
B220
CD19
CD8a
CD3ε
CD4
Nkp46
FcεR1a
0
2e+05
4e+05
6e+05
n cells
Mouse id
Scaled mean expression
Normalized frequency
Ctrl
Ctrl
Ctrl
HSCT
HSCT
HSCT
(E)
Related proportion in BM cells
1.5
1.0
0.5
0.0
NK cell
DC
Ctrl
HSCT-4M
(F)
Related count in BM cells
0.888
0.310
0.651
0.700
0.053
0.554
0.805
0.343
0.030
0.613
0.026
0.247
0.820
Granulocyte
B cell
Lin- cell
Monocyte
Dendritic cell
Treg cell
CD8 T cell
HPC
NK cell
Mast cell
Macrophage
Epithelial cell
CD4 T cell
Related to Ctrl group
Ctrl
HSCT-4M

Figure S3

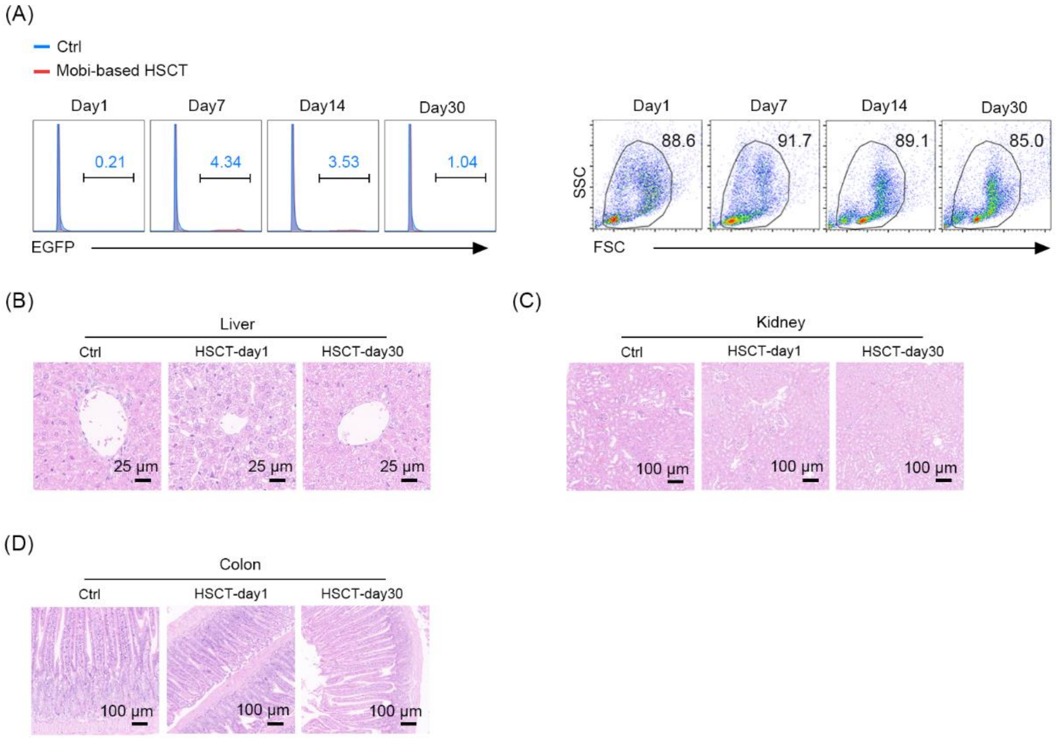
(A)
Ctrl
Mobi-based HSCT
Day1
Day7
Day14
Day30
0.21
4.34
3.53
1.04
EGFP
88.6
91.7
89.1
85.0
SSC
FSC
(B)
Liver
Ctrl
HSCT-day1
HSCT-day30
25 µm
(C)
Kidney
100 µm
(D)
Colon

Figure S4

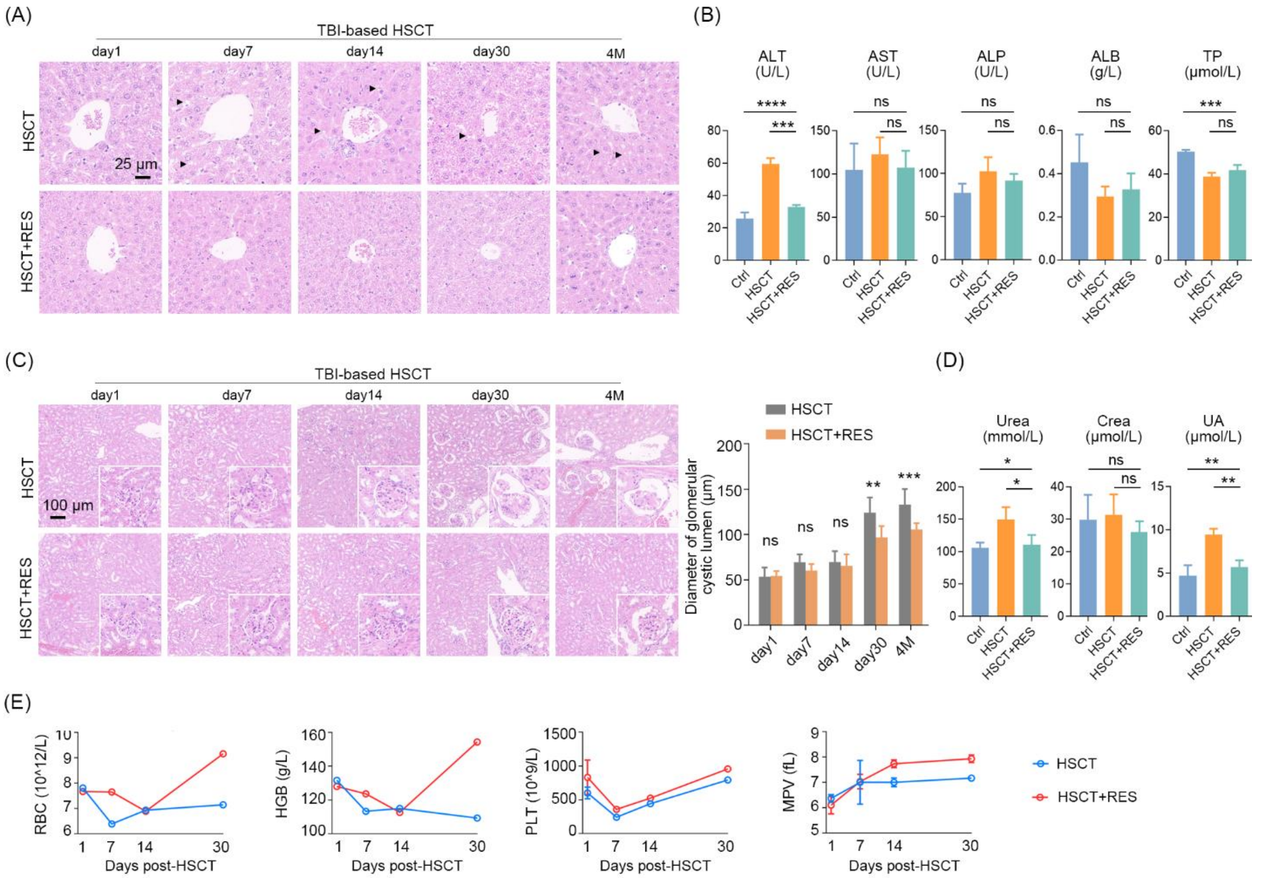
(A)
TBI-based HSCT
day1
day7
day14
day30
4M
HSCT
HSCT+RES
25 μm
(B)
ALT (U/L)
AST (U/L)
ALP (U/L)
ALB (g/L)
TP (μmol/L)
Ctrl
HSCT
HSCT+RES
(C)
TBI-based HSCT
100 μm
HSCT
HSCT+RES
Diameter of glomerular cystic lumen (μm)
(D)
Urea (mmol/L)
Crea (μmol/L)
UA (μmol/L)
(E)
RBC (10^12/L)
HGB (g/L)
PLT (10^9/L)
MPV (fL)
Days post-HSCT
HSCT
HSCT+RES

SUPPLEMENTARY FIGURE LEGENDS

Figure S1 Hematopoietic stem cell transplantation enables hematopoietic reconstitution in mice. **A** Schematics of operation and validation used in the study. **B** Results (left) and percentage (right) of Y-chromosome fragment probe (ChrY-Texas-red) on bone marrow smears using DNA fluorescence in situ hybridization in ctrl and HSCT mice. **C** qRT-PCR analysis illustrating the mRNA levels of *Sry* gene in bone marrow cells. **D** Flow cytometry determining the percentage of chimeras (EGFP$^+$ cells) in bone marrow (left), peripheral blood monocular cells (PBMC; middle), and spleen (right). **E** CFU assay of HSPCs derived from the bone marrow of ctrl and HSCT mice on the day 30 following HSCT. ns (no significance) $p > 0.05$, *$p < 0.05$, **$p < 0.01$, ***$p < 0.001$, ****$p < 0.0001$

Figure S2 CyTOF analysis of immune cells from the ctrl and HCT mice at the first, second and the fourth month post-transplantation. **A–B** UMAP plots illustrating the dimension reduction results of 182,747 bone marrow cells (A) and 210,370 spleen cells (B). The cells were grouped by cell type (left) and condition (right). **C** UMAP plot of 1,299,324 bone marrow cells from ctrl and SHCT mice at the fourth month post transplantation. **D** Feature plot illustrating the marker expression (left), cell composition (middle), and cluster density (right) of the respective mice mentioned above. **E** Quantitative differences in counts of various cell types in the ctrl and fourth-month post-transplantation mice. **F** Related bar plot showing related cell counts of the ctrl and the fourth month post-transplantation mice.

Figure S3 Mobilization-based transplantation does not result in radiation-induced damage. **A** Flow cytometry depicting the proportion of $EGFP^+$ cells (left) and effective cells (right) within bone marrow from ctrl and mobilization-based HSCT mice on day 1, day 7, day 14 and day 30 post-transplantation. **B–D** HE staining of liver, kidney and intestine of ctrl and mobilization-based HSCT mice.

Figure S4 Resveratrol mitigates the hepatotoxicity and nephrotoxicity induced by TBI. **A** HE staining of liver in HSCT and HSCT-RES mice on day 1, day 7, day 14, day 30, and 4-month post-transplantation. **B** Levels of liver function indices. **C** HE staining of kidney, and diameters of glomerular cystic lumen of the above-mentioned groups. **D** Levels of kidney function indices. **E** Whole blood count of HSCT and HSCT-RES mice on day 1, day 7, day 14 and day 30 post-transplantation.

Table S1 CyTOF antibodies and regents

Target	Tag	Source	CAS No.
CD45	89Y	Standard BioTools	#3089005B
B220	106Cd	R&d	#MAB1217
BST-2	116Cd	Standard BioTools	#MAB8660
IL-3R	140Ce	R&d	#MAB983
CD205	142Nd	Abcam	#ab208649
CD66a	143Nd	R&d	#AF6480
FcεRIIA	144Nd	R&d	#MAB6900
CD4	145Nd	Standard BioTools	#3145002B
F4/80	146Nd	Standard BioTools	#3146008B
ST2	147Nd	R&d	#MAB10041
CD95	148Nd	Abcam	#ab227907
CD19	149Sm	Standard BioTools	#3149002B
CCR7	150Nd	R&d	#MAB3477
CD49d	151Eu	Standard BioTools	#3151016B
CD3ε	152Sm	Standard BioTools	#3152004B
Nkp46	153Eu	Standard BioTools	#3153006B
CTLA-4	154Sm	Standard BioTools	#3154008B
CD138	155Gd	R&d	#MAB2966
PD-1	159Tb	Standard BioTools	#3159006B
CD62L	160Gd	Standard BioTools	#3160008B
Ly-6C	162Dy	Standard BioTools	#3162014B

CD38	163Dy	R&d	#AF4947
Sca-1	164Dy	Standard BioTools	#3164005B
CD326	165Ho	Standard BioTools	#3165014B
CD117	166Er	Standard BioTools	#3166004B
CXCR3	167Er	R&d	#MAB1685
CD8a	168Er	Standard BioTools	#3168003B
CD206	169Tm	Standard BioTools	#3169021B
IL-7RA	170Er	R&d	#AF747
CD80	171Yb	Standard BioTools	#3171008B
CD11b	172Yb	Standard BioTools	#3172012B
CD21	173Yb	Abcam	#ab271855
IL-17RE	174Yb	R&d	#MAB7997
FcεR1A	176Yb	Standard BioTools	#3176006B
CD11c	209Bi	Standard BioTools	#3209005B
CD16/CD32 Fc block		BD	#553142
Cell Staining Buffer		Standard BioTools	#201068
Cell Acquisition Buffer		Standard BioTools	#201240
Fix and Perm Buffer		Standard BioTools	#201067
EQ Four Elements Calibration Beads		Standard BioTools	#P20C3116
Cell-ID Cisplatin	195Pt	Standard BioTools	#201064
Cell-ID Intercalator-Ir	191Ir/193Ir	Standard BioTools	#201192B
